# The impact of myocardial infarction on basal and stress-induced heart rate variability and cortisol secretion in women: A pilot study

**DOI:** 10.1016/j.cpnec.2022.100113

**Published:** 2022-01-13

**Authors:** N.F. Narvaez Linares, K. Munelith-Souksanh, A.F.N. Tanguay, H. Plamondon

**Affiliations:** a Behavioural Neuroscience Group, School of Psychology, University of Ottawa, 136 Jean-Jacques Lussier, Ottawa, On, K1N 6N5, Canada; b Faculty of Science, University of Ottawa, 30 Marie-Curie, Ottawa, On, K1N 6N5, Canada; c Integrated Neurocognitive & Social Psychophysiology Interdisciplinary Research Environment (INSPIRE) Laboratory, Faculty of Social Sciences, University of Ottawa, 136 Jean-Jacques Lussier, Ottawa, On, K1N 6N5, Canada

**Keywords:** Myocardial infarction, Women, Trier social stress test, Cortisol, Heart rate, Perceived stress

## Abstract

Coronary heart disease (CHD), of which myocardial infarction (MI) is a subtype, is the leading cause of death for women. Nonetheless, women remain neglected in CHD research, resulting in treatments and recommendations being primarily based on data collected in men. Pre-clinical and clinical studies have supported dysregulation of the hypothalamic-pituitary-adrenal axis (HPAA) following cardiac arrest and MI to promote the development of mental health disorders (e.g., major depressive disorder, post-traumatic stress disorder). However, studies addressing changes in HPAA activation under basal and stress-induced conditions in women samples have been lacking. Thus, we conducted this study to determine basal and stress-induced changes in heart rate, respiration and cortisol secretion (via 8 saliva samples) in a sample of women with a history of MI (*n* = 13) and a control group (*n* = 16). We measured altered stress reactivity through exposure to the Trier Social Stress Test. In addition, participants completed questionnaires assessing perceived stress and mental health status (i.e., anxiety and mood). Overall, our findings indicated comparable assessments of perceived situational stress in both groups. Interestingly, salivary cortisol secretion support reduced stress-induced HPAA activation related to TSST exposure in MI women compared to control counterparts. Our observations are consistent with findings supporting glucocorticoid resistance noted following MI and cardiac arrest. Akin to cardiac arrest survivors, HPAA dysregulation in MI survivors could have an impact on the development of mental health disorders. More studies are needed to address this critical question.

## Highlights

• Women have been neglected in coronary heart disease for decades.
• Little is known regarding the impact of myocardial infarction on physiological markers of the HPAA (e.g., cortisol).
• Women with a history of MI perceived similar levels of stress compared to the control group, but CORT secretion was altered.
• Physiological results support MI to affect HPAA function upon stress, which could contribute to increased mental health issues.

## Abbreviations

AUC_G_: Area under the curve to ground
AUC_I_: Area under the curve to increase
CHD: Coronary heart disease
CVD: Coronary vascular disease
HPAA: Hypothalamic-pituitary-adrenal axis
HRV: Heart rate variability
MI: Myocardial Infarction
PANAS: Positive and Negative Affect Schedule
STAI: State-Trait Anxiety Inventory
SCA: Sudden cardiac arrest
TSST: Trier Social Stress Test
RSA: Respiratory sinus arrhythmia

## 1 Introduction

Coronary heart disease (CHD), also known as ischemic heart disease or coronary artery disease, is the leading cause of death and the most prevalent subtype of cardiovascular disease (CVD) worldwide [1]. A CHD develops from atherosclerosis due to a lack of oxygen-rich blood supply to the heart related to partial or complete occlusion of the arteries' walls. Myocardial Infarction (MI), a subtype of CHD, is amongst the most prevalent CVD, killing one person every 40 s in the United States [1]. Worldwide, MI and stroke account for 85% of all deaths caused by CVD [2].

Unfortunately, women^2^ have been neglected from scientific and medical research for decades [3,4], and the field of CVD has not been spared [[5], [6], [7]]. Women have been systematically excluded or recruited at lower levels than men [4,8]. Indeed, two-thirds of heart disease research samples continue to predominately include men [9]. The lack of women inclusion significantly impacts health care practitioners' levels of knowledge and the development of specific treatment guidelines [1,10]. For example, only 22% of family doctors and 42% of cardiologists report being qualified to assess the risk of heart disease in women [11]. Women have a 30% increased likelihood to die from an MI compared to men [12], and if the attending physician is a man, the risk of death tends to increase compared with having a woman attending physician [13]. Women tend to experience different symptoms (e.g., back pain, cold sweats) when experiencing an MI [14], which are considered 'atypical' and are not well known [15,16]. In this context, both the scientific community and the general population remain to be properly educated on sex-specific symptomatology. The paucity of knowledge reflects on the consequences of MI in women.

### 1.1 Psychological impact of myocardial infarction

In women, the risk of mental health disorders such as anxiety and depression increases significantly after an MI [17,18]. Importantly, people diagnosed with a major depressive episode following MI are more likely to die [19,20]. The latter is also true for anxiety disorders [21,22]. Compared to men, women tend to experience reduced psychological well-being and increased psychological distress following an MI, even five years following the event [23]. Overall, the levels of perceived stress following an MI are heightened in women [24], who tend to present worsened recovery prognostics and report more posttraumatic stress symptoms [[25], [26], [27]]. In other words, the impact of MI extends beyond impaired physical capacities.

### 1.2 Physiological impact of myocardial infarction

A bi-directional relationship exists between stress and MI, with stress exposure increasing the risk of CHD and MI occurrence [[28], [29], [30], [31], [32]]. Considering the negative impact of stress on post-infarct recovery [[30], [105], [106], [107]], changes in hypothalamic-pituitary-adrenal axis (HPAA) activation following MI warrant further investigation. Responsible for the body's neuroendocrine response to stress, its response is initiated by the secretion of corticotropin-releasing factor from the hypothalamus, which is followed by the release of the adrenocorticotropic hormone by the anterior pituitary gland, resulting in glucocorticoid release by the adrenal glands, which ultimately acts on cardiovascular functions [33]. Researchers have found that a sustained increase in endogenous glucocorticoid levels is linked to cardiovascular complications such as systemic arterial hypertension and metabolic syndrome [34,35].

The dysregulation of the HPAA following sudden cardiac arrest (SCA) has been studied for years [36,37]. Whereas SCA is described as the sudden malfunctioning of the electrical system of the myocardium [38], MI is the result of an occluded artery inducing a state of hypoxia and ultimately resulting in different levels of necrosis in the heart muscle [39]. Hypoxia-induced by SCA and MI events acts as a metabolic stressor (e.g., HPAA dysregulation). Research suggests HPAA dysregulation to stem from hippocampal damage and adrenal insufficiency [[40], [41], [42]]. For example, Neigh et al. [43] demonstrated blunted stress-induced cortisol secretion two weeks following SCA in mice, associated with hippocampal neuronal damage. Apart from neuronal brain damage, there is evidence that the adrenal gland - a key player in the HPAA-is also impacted. In the event of an SCA, the body becomes suddenly anoxic, and the concentration of epinephrine increases; this can cause varying degrees of necrosis to the adrenal gland [43]. As a result, the HPAA integrity is compromised and blunted cortisol concentration is observed in response to stress [44,45]. The lack of brain oxygenation and nutriments, which characterize MI and 10.13039/501100000148SCA, could support common physiological impairments. Supporting this contention, Kaplan et al. [46] have reported reduced cerebral blood flow up to 30 days post-MI, which renders plausible the contribution of brain injury in MI-induced effects on HPAA and cognitive functions.

There is no doubt that stress impacts both physiological and mental states [108]. Psychological - measured with tests - and physiological responses - measured with biomarkers such as cortisol - exist as indicators of the stress construct, and a strong association between these two types of reactions is present in healthy individuals [110], and exemplified in controlled laboratory environments through changes in both affective and physiological states when psychosocial pressures appear [111]. For example, Oldehinkel et al. [109] found subjective reports of arousal and unpleasantness in a sample of Dutch adolescents (*N* = 715) to be related to respiratory sinus arrhythmia (RSA) and cortisol responses during the performance of a stressful task. This interaction between psychological and physiological responses coincides with a substantial overlap in their neural pathways (e.g., projections from the hippocampus and amygdala; [[47], [48], [49]]. Additionally, all regions within this system express glucocorticoid receptors and contribute to HPAA regulation of psychophysiological responses [50]. In other words, psychological and physiological responses are highly associated, as this is evident through scientific experimentation and the structural cortical supports in the human brain.

### 1.3 Study objectives

The main goal of this pilot research was to compare HPAA activation in women having experienced an MI and an age-matched control group before and after a stressful task. The physiological response was determined using different biomarkers (i.e., cortisol, heart rate variability). In addition, the levels of perceived stress were evaluated at different time intervals to assess the impact of the stressful task.

## 2 Method

### 2.1 Participant characteristics

Participants were recruited from an online study by indicating their interest in an in-person study. The following information was extracted from the online study to compare the difference between the MI and the control (Non-MI) groups: a) age; b) body mass index (BMI); c) marital status; d) level of education; e) household income; f) employment status; g) ethnic group; h) menopause status; i) status of reproductive organs (i.e., if were removed and when); j) MI information (i.e., number); k) CHD information; l) received diagnoses of high blood pressure, high cholesterol, and diabetes; m) prescribed medication; and n) language of study.

### 2.2 Sampling procedures

We included participants if: a) aged between 45 and 80; b) no current or past substance use; c) no neurological condition or been diagnosed with dementia; d) no current psychiatric disorder; e) were not taking the contraceptive pill; f) were not following a hormonal replacement therapy; g) if still menstruating, their cycle needed to be regular (between 21 and 35 days); and h) were not pregnant or breastfeeding at the time. For participants that indicated having a MI condition, the diagnoses needed to be made by a physician. We conducted the study between 9 a.m. and 12 p.m. Therefore, to control for possible external factors that could influence the measurement of their HPAA response, after 8 a.m., participants were instructed to not: a) consume any alcohol and tobacco; b) ingest any food or drink any caffeinated beverage; c) floss or brush their teeth; and d) engage in moderate or high-intensity physical activity.

Participants were from the Ottawa-Gatineau region in Canada and could complete the study in French or English. Free parking was provided in one of the university parking lots, and they were given a choice to pick a 15$ gift card from different stores. When participants were scheduled, only one of the principal investigators (N.F.N.L.) was aware of their status (MI or Non-MI). The ethics board of the University of Ottawa approved the ethical aspect of this study (H-06-18-639).

### 2.3 Sample size, power, and precision

The study initiated in August 2019 ended in March 2020 due to the global pandemic. This led to the recruitment of a limited number of participants, with restricted possibilities to pursue post-pandemic assessments under similar basal conditions. A total of twenty-nine women were tested [*N* = 29 (n_*MI*_ = 13; *n*_*NonMI*_ = 16)]. Of thirty-four participants initially recruited, 5 were excluded; three participants arrived too late, and we could not take physiological measures, one participant had a major eye surgery less than a year ago, and the software for one participant did not properly record data. We used G*Power 3.1.9.7 [51] and found a power of 0.99 to detect a within-between interaction with a sample size of 29 with the following parameters: a) estimated effect size of *η*_*p*_^*2*^ = 0.10; b) α = 0.05; c) number of groups = 2; d) number of measurements = 8, e) correlation among repeated measures = .5 (default), and d) nonsphericity correction = 1 (default).

### 2.4 Data collection

**Fig. 1:**
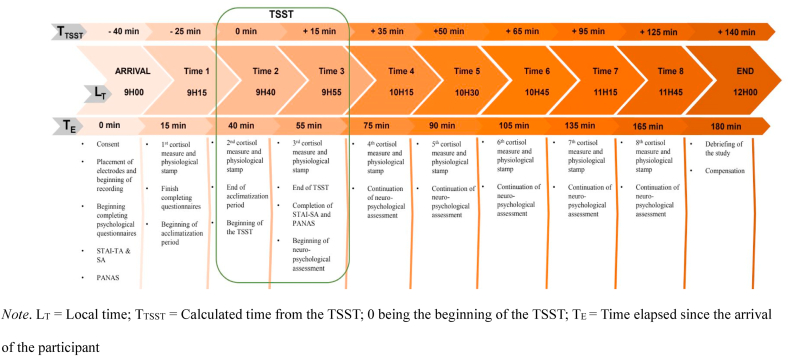
Timeline of the study *Note*. L_T_ = Local time; T_TSST_ = Calculated time from the TSST; 0 being the beginning of the TSST; T_E_ = Time elapsed since the arrival of the participant.

All participants were invited for a 3-h study in the Integrated Neurocognitive & Social Psychophysiology Interdisciplinary Research Environment Laboratory at the University of Ottawa. Research assistants in charge of collecting the physiological measures were blind to the assigned participants' group; Fig. 1 shows a detailed timeline of the study. In brief, participants were accompanied from the parking lot to the laboratory. Consent to participate was obtained, an explanation of the study was provided, and six electrodes were placed on the upper part of their body to collect physiological measures for the entire study duration. Then, participants completed a series of psychological questionnaires. Following this, they were given a choice to read a book they brought – had to be a non-anxiety-provoking book – or watch a safari documentary [52]. Participants then completed the Trier Social Stress Test (TSST). Following TSST exposure, two psychological questionnaires assessed the participants' perceived stress, and they completed a series of neuropsychological tests. As the study ended, participants completed the same two psychological tests and received a debriefing. Saliva samples were collected at 8 key experimental intervals using the SalivaBio Passive Drool Method from Salimetrics®. For more details about the measures, please see section 2.5.

### 2.5 Measures

#### 2.5.1 Trier Social Stress Test

The TSST was created to objectively study HPAA changes by combining two inducing stressors [53]. Since its creation, the TSST has been widely used in more than 1000 peer-reviewed studies [54] and for most participants (70–80%), there is a rise of salivary cortisol levels up to threefold [55]. Our laboratory performed an extensive systematic review of TSST methodology because researchers have not applied the method consistently since its creation, which could influence HPAA activation, warranting careful consideration when interpreting findings made with this task [54]. We applied the guidelines provided in this review. For more details about the administration of the TSST, please see the Supplementary Material document.

#### 2.5.2 Psychological tests

##### 2.5.2.1 State-Trait Anxiety inventory (STAI)

The STAI is a self-reported measure made of 40 items on a 4-point Likert scale that allows assessing trait (20 items; STAI-TA) – how they feel in general; and state (20 items; STAI-SA) – how they feel in the present moment [56]. The maximum is 40 on each scale; a higher score indicates higher anxiety levels. The STAI has good reliability and validity and is widely used across studies because it is available in many languages and is simple to administer [56,57]. Our participants completed the STAI-TA at T_1_ and the STAI-SA at T_1_, T_3_, and T_8_.

##### 2.5.2.2 Positive and negative affect schedule (PANAS)

The PANAS is a self-reported measure made of 10 items, equally divided to measure positive and negative affects. The participant must rate on a 5-point Likert scale to what extent they feel the described emotion at the present moment [58]. The higher the score, the higher the participant feels this emotion (negative or positive), the maximum score being 50. The PANAS's validity and reliability have been strongly rated [58,59], and the test has been used in several studies, being simple to administer and available in different languages. Our participants completed the PANAS at T_1_, T_3_, and T_8_.

#### 2.5.3 Physiological measures

##### 2.5.3.1 Unbound cortisol

Saliva samples were collected in Eppendorf tubess and placed immediately on ice after collection. Any deviations in a participant's saliva collection time were recorded, accompanied by an explanation. All samples were stored in −80 °C freezers until concentrations were later determined. The unbound cortisol concentration was determined using the Salimetrics Expanded Range High Sensitivity Salivary Cortisol Enzyme Immunoassay Kit (ELISA), as recommended by the manufacturer [60]. All cortisol samples were run in duplicates. The plates were read at 450 nm using the BioTek PowerWave XS, and BioTek Gen5 was used to determine the coefficients of variability (CV). The inter-assay CV is 8.230, and the intra-assay CV is 5.89, which both meet the acceptable CVs set by Salimetrics [55].

##### 2.5.3.2 Heart rate variability

Heart rate variability (HRV) refers to the fluctuations in cardiac rhythm and is a common measure used to represent the physiological response of the autonomic nervous system [61], precisely the sympathovagal balance at the sinoatrial level [62,63]. Our study recorded the HRV activity using a non-invasive electrocardiogram technique, and focused on analyzing one type of HRV measure called respiratory sinus arrhythmia (RSA) – defined as the natural logarithm of high-frequency power [64]. The RSA metric represents the change in heart rate as a function of respiration, which falls within the high-frequency range [65]. The RSA metric indexes [65]. The RSA metric indexes regulation of the parasympathetic system and higher values reflect greater parasympathetic control.

We measured HRV with an electrocardiogram, using pre-gelled Ag/AgCl sensors in a modified lead II configuration. We used Mindware Technologies BioLab v.3.0.13 through a BioNex 8-slot chassis (Model 50-3711-08) with a sampling frequency of 1000 Hz to acquire our data. We analyzed HRV data in Mindware Analysis Application Version 3.2.9, released March 30, 2021. We applied a 60 Hz notch filter and a bandpass filter between 0.25 Hz and 45 Hz to reduce noise due to electrical interference or movement. We derived the respiration signal from the impedance cardiography signal. Noise compromised the accurate identification of B points in the impedance cardiography signal, and these data were not considered further. Please see the Supplementary Material document for more information about how the data were inspected and analyzed.

### 2.6 Statistical analyses

We carried out ANOVAs and t-tests in IBM SPSS Statistics 28 [66], and repeated measures correlations in R 1.4.1 [67] using the rmcorr package [68,69]. We assessed data distribution and assumptions for all variables depending on analysis. A root square transform solved normality issues for cortisol measurement, but PANAS scores could not be converted to normality. All other measures were normally distributed. Due to the small MI group (<15), we screened for outliers in ungrouped data and defined outliers as z ± 2.00. We replaced outliers with the corresponding value at z ± 2.00. We applied a Greenhouse-Geisser correction [70] when repeated measures did not meet the assumption of sphericity. The p-level was set at 0.05 for all analyses, and tests were two-tailed. We elaborated a detailed section for the statistical analyses that can be found in the Supplementary Material document.

## 3 Results

### 3.1 Sociodemographic

The characteristics of our participants can be found in Table 1, and information related to medication can be found as supplementary material. The average age of our participants was 56.92 (*SD* = 8.59) and 61.44 (*SD* = 9.21) for the MI and NoMI groups, respectively. The average BMI was 27.47 (*SD* = 4.12) and 25.75 (*SD* = 3.27) for the MI and NoMI groups, respectively. Demographic and medical characteristics were compared between women with and without a history of MI to establish a clearer profile of women with and without a history of MI. Welch independent samples *t*-test found women with MI took significantly longer to complete the study than women without a history of MI, *t*(17.72) = 3.12, *p* = .006, *d* = 1.23. Independent samples *t*-test revealed a tendency for women with a history of MI to have a higher BMI than women without a history of MI, *t*(25) = 1.91, *p* = .068, *d* = 0.74. Women with MI reported more hypertension diagnoses than women without MI [χ^2^ (1) = 3.95, *p* = .047], and Fisher's exact test revealed women with MI tended to report more Type II diabetes, *p* = .064. We did not find other group differences for the remaining demographic variables.

**Table 1:** Participant characteristics.

Characteristics	N	n_MI_	n_NoMI_
Language of survey			
English	26	13	13
French	3	0	3
Age group			
35–44	0	0	0
45–54	11	6	5
55–64	5	4	1
65–74	13	3	10
75–84	0	0	0
Ethnicity			
White	26	11	15
Arab or West Asian	1	1	0
Chinese	1	1	0
Preferred not to answer this question	1	0	1
BMI (Kg/m^2^)			
Healthy weight (18.5–24.9)	12	4	8
Pre-obesity (25.0–29.9)	11	6	5
Obesity class I (30.0–34.9)	3	2	1
Obesity class II (35.0–39.9)	0	0	0
Obesity class III (>40)	1	1	0
Preferred not to answer this question	2	0	2
Marital status			
Currently married	18	8	10
Divorced	2	2	0
Common-law or in a relationship	6	3	3
Single	1	0	1
Widowed	2	0	2
Separated	0	0	0
Education level			
No certificate, diploma, or degree	0	0	0
Secondary (high) school diploma or equivalency certificate	3	3	0
Certificate of apprenticeship, certificate of Qualification, or Trades certificate	0	0	0
College, Cégep, or other non-university certificate diploma	9	4	5
University certificate or diploma below bachelor level	1	0	1
Bachelor's degree	8	3	5
Professional degree (e.g., MD)	2	0	2
Master's degree	6	3	3
Earned Doctorate	0	0	0
Household income (CAD $)			
<19,999	0	0	0
20,000–29,999	0	0	0
30,000–39,999	2	2	0
40,000–49,999	1	0	1
50,000–59,999	1	0	1
60,000–69,999	1	1	0
70,000–79,999	2	1	1
80,000–89,999	2	1	1
90,000–99,999	5	2	3
+100,000	12	6	6
Preferred not to answer the question	3	0	3
Employment			
Full-time employee	6	3	3
Part-time employee	3	2	1
Self-employed	4	3	1
Unemployed or retired	13	4	9
On social welfare	0	0	0
Type of contraception			
No contraceptive	29	13	16
Oral contraceptive pill	0	0	0
Contraceptive patch	0	0	0
Vaginal ring	0	0	0
Intrauterine contraception	0	0	0
Injectable contraception	0	0	0
Hormonal replacement therapy			
No	29	13	16
Yes	0	0	0
Menopause			
No	10	5	5
Yes	19	8	11
Type of surgery (reproductive organs)			
"I had a bilateral oophorectomy"	1	0	1
"I had a hysterectomy, with or without oophorectomy"	3	1	2
No surgery	25	12	13
If surgery			
Before their menopause	2	0	2
During their menopause	1	1	0
After their menopause	1	0	1
Pregnancy			
No	3	1	2
Yes	26	12	14
Number of MI			
1	–	8	–
2	–	4	–
3	–	1	–
Diagnosis of CHD			
No	21	8	13
Yes	8	5	3
Diagnosis of hypertension			
No	17	5	12
Yes	12	8	4
Diagnosis of high cholesterol			
No	19	9	10
Yes	7	4	3
I do not know	3	0	3
Diagnosis of Diabetes types 2			
No	23	8	15
Yes	6	5	1

### 3.2 Psychological impact of stress

#### 3.2.1 PANAS

We entered the PANAS scores in a mixed ANOVA with Valence (positive, negative) as a within-subject factor, Time (T_1_, T_3_, T_8_) as a within-subject factor, and Group as a between-subject factor (NoMI, MI). The main effect of Time was significant, *F*(2, 54) = 6.47, *p* = .003, *η*_p_^2^ = 0.19, and Valence was significant, *F*(1, 27) = 255.44, *p* < .001, η_p_^2^ = 0.90. The effect of Valence depended on Time, *F*(2, 54) = 20.35, *p* < .001, η_p_^2^ = 0.43.

**Fig. 2:**
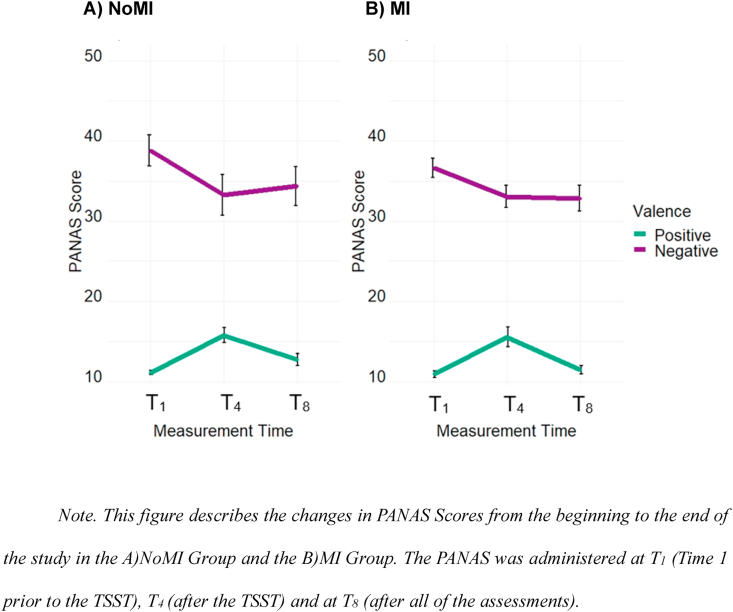
Change in PANAS Scores throughout the study period Note. This figure describes the changes in PANAS Scores from the beginning to the end of the study in the A)NoMI Group and the B)MI Group. The PANAS was administered at T_1_ (Time 1 prior to the TSST), T_4_ (after the TSST) and at T_8_ (after all of the assessments).

Positive affect increased from T_1_ (*M* = 10.90, *SE* = 0.18) to T_3_ (*M* = 15.64, *SE* = 0.76), *p* < .001 (see Fig. 2). Positive affect decreased from T_3_ to T_8_ (*M* = 11.96, *SE* = 0.41), *p* < .001. Positive affect was, nevertheless, still higher at T_8_ than T_1_, *p* = .015. Negative affect decreased from T_1_ (*M* = 37.71, *SE* = 1.21) to T_3_ (*M* = 33.10, *SE* = 1.52), *p* < .001, and it was also lower at T_8_ (*M* = 33.54, *SE* = 1.31; than T_1_), *p* = .001. T_3_ and T_8_ did not differ in negative affect, *p* = .64. None of these effects depended on Group: Time*Group, *F*(2, 54) = 0.80, *p* = .45, η_p_^2^ = 0.03; Valence*Group, *F*(1, 27) = 0.06, *p* = .81, η_p_^2^ = 0.002, Time*Valence*Group, *F*(2, 54) = 0.25, *p* = .78, η_p_^2^ = 0.01. There was no main effect of Group, *F*(1, 27) = 0.60, *p* = .44, η_p_^2^ = 0.02.

#### 3.2.2 STAI

**Fig. 3:**
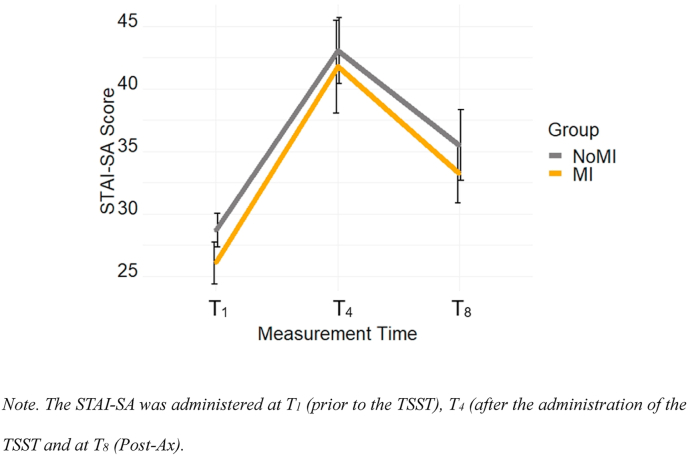
Change in STAI-SA Scores throughout the study period Note. The STAI-SA was administered at T_1_ (prior to the TSST), T_4_ (after the administration of the TSST and at T_8_ (Post-Ax).

3.2.2.1 STAI - SA. The STAI-SA score was entered in a mixed ANOVA with Time (T_1_, T_3_, T_8_) as a within-subject factor and Group (NoMI, MI) as a between-subject factor. The effect of Time was significant, *F*(2, 54) = 36.48, *p* < .001, η_p_^2^ = 0.58, and did not depend on Group, *F*(2, 54) = 0.06, *p* = .95, η_p_^2^ = 0.002. The main effect of Group was also not significant, *F*(1, 27) = 0.48, *p* = .49, η_p_^2^ = 0.02. Participants felt higher levels of increased anxiety from T_1_ (*M* = 27.38, *SE* = 1.06) to T_3_ (*M* = 42.27, SE = 2.15), *p* < .001, and scores were still higher at T_8_ compared to T_1_ (*M* = 34.05, *SE* = 1.78), *p* < .001 (see Fig. 3). Scores decreased from T_3_ to theT_8_, *p* < .001.

##### 3.2.2.1 STAI - TA

**Fig. 4:**
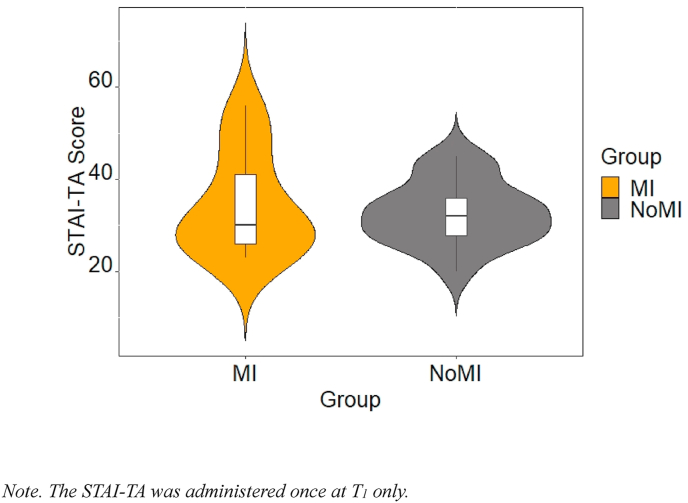
STAI-TA Scores as a function of the study groups Note. The STAI-TA was administered once at T_1_ only.

An independent samples *t*-test showed that the NoMI group (*M* = 32.69, *SE* = 1.71) and the MI group (*M* = 33.24, *SE* = 2.43) were not significantly different on the STAI-TA (see Fig. 4), mean difference of −0.56, BCa 95% [−6.44, 5.05], *t*(27) = −0.19, *p* = .85, Hedges’ *g* = −0.07, CI 95% [−0.78, 0.64].

### 3.3 Physiological reaction to stress

#### 3.3.1 Cortisol

Cortisol values were analyzed using a mixed ANOVA with Group (MI, NoMI) as a between-subject factor, and Time (T_1_ toT_8_) as a within-subject factor. The main effect of Time was significant [*F*(4.15, 107.98) = 7.51, *p* < .001, η_p_^2^ = 22]. Specifically, the highest cortisol values were observed at T_1_, presumably due to the ongoing acclimatization to the laboratory environment, and at T_4_, as expected due to the TSST exposure.

Only significant results are presented; Table 2 provides a summary of all *p*-values. T_1_ was significantly higher than T_2_ (*p* = .016), T_3_ (*p* = .017), T_6_ (*p* = .001), T_7_ (*p* < .001), T_8_ (*p* < .001). T_4_ was significantly higher than T_6_ (*p* < .001), T_7_ (*p* < .001) and T_8_ (*p* < .001). Cortisol decreased gradually after T4; hence, T_7_ had significant lower cortisol values than T_2_ (*p* = .016), T_3_ (*p* = .018), and T_5_ (*p* < .001). T_8_ also had significant lower cortisol values than T_3_ (*p* = .043), and T_5_ (*p* = .002). Lastly, T_6_ had a significant lower cortisol value than T_5_ (*p* = .002). Contrary to expectation, the main effect of Group was not significant, *F*(1, 26) = 1.78, *p* = .19, η_p_^2^ = 0.06, and there was no interaction between Group and Time, *F*(4.15, 107.98) = 0.93, *p* = .48, η_p_^2^ = 0.04.

**Table 2:** Difference between times for cortisol measures (p-values).

	T_2_	T_3_	T_4_	T_5_	T_6_	T_7_	T_8_
T_1_	**.16**	**.017**	.38	.055	**.001**	< **.001**	< **.001**
T_2_		.91	.13	.67	.11	**.016**	.058
T_3_			.10	.64	.086	**.018**	**.043**
T_4_				.12	< **.001**	< **.001**	< **.001**
T_5_					**.002**	< **.001**	**.002**
T_6_						.16	.33
T_7_							.92

*Note.* Bold p-values indicates a significant result (<0.05).

**Fig. 5:**
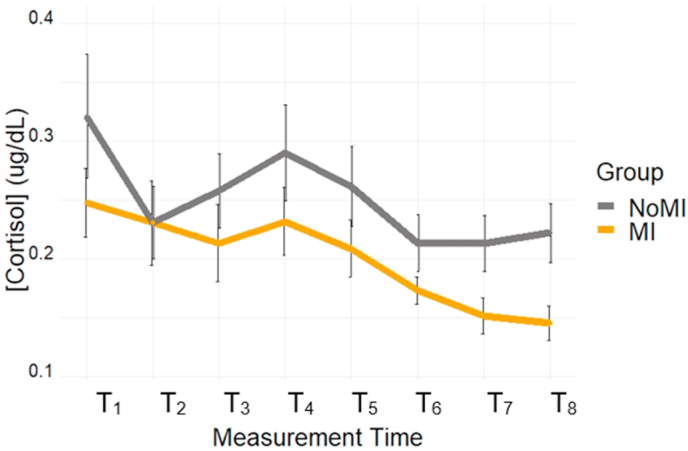
Salivary Cortisol Concentration throughout the study.

**Fig. 6:**
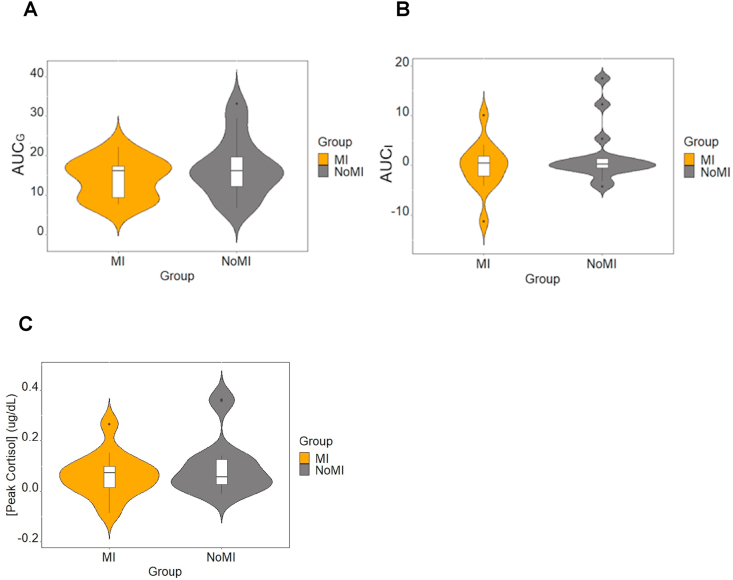
A)AUC_G_, B)AUC_I_, and C) Peak Reactivity compared between the study groups.

To fully characterize the cortisol response profile, targeted analyses of the TSST period were performed using peak reactivity and the area under the curve to ground (AUC_G_) and to increase (AUC_I_), comparing the two groups (see Fig. 5). No group differences were found on AUC_G_, AUC_I_, and peak reactivity (see Fig. 6). For more details on these analyses, see the Supplementary Material document.

Based on a priori hypotheses, independent t-tests compared the groups at each time interval (excluding the participant without a measurement at T_1_ for consistency). Our findings showed that the MI group (*M* = 0.38, *SE* = 0.02) showed reduced cortisol values at T_8_ compared to the NoMI group (*M* = 0.46, *SE* = 0.02); mean difference of 0.08, BCa 95% [0.02, 0.14], *t*(26) = 2.68, *p* = .015, Hedges' *g* = 0.92, CI 95% [0.14, 1.68]. A statistical trend suggests that the difference started emerging earlier: at T_7_, the MI group (*M* = 0.38, *SE* = 0.02) showed reduced cortisol values compared to the NoMI group (*M* = 0.45, *SE* = 0.03); mean difference of 0.07, BCa 95% [0.01, 0.12], *t*(26) = 2.00, *p* = .062, Hedges’ *g* = 0.69, CI 95% [−0.06, 1.44]. We did not find a significant (or near significant) difference for time points prior to T_7_.

#### 3.3.2 Heart rate variability

**Fig. 7:**
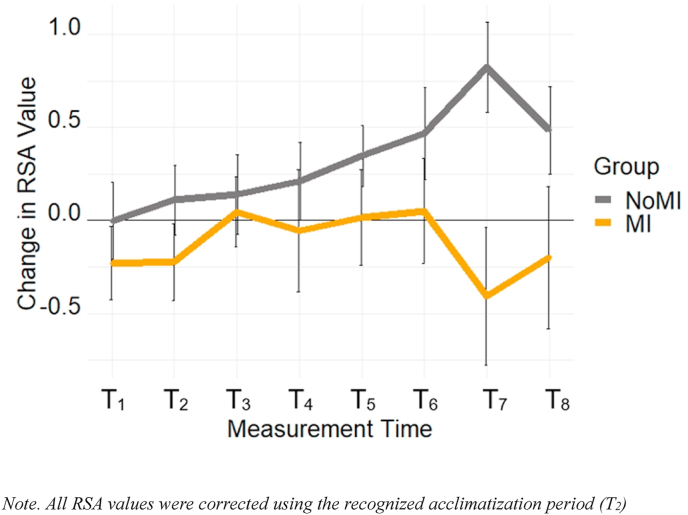
Change in RSA Value throughout the study period Note. All RSA values were corrected using the recognized acclimatization period (T_2_).

A mixed ANOVA was conducted with Group (NoMI, MI) and the eight selected time intervals (T_1_ to T_8_), using RSA as the dependent variable (see Fig. 7). Analyses revealed no main effects of Group, *F*(1, 24) = 2.21, *p* = .15, η_p_^2^ = 0.08 and Time, *F*(4.50, 108.03) = 1.95, *p* = .10, η_p_^2^ = 0.08, and also for no Group by Time interaction *F*(4.50, 108.03) = 2.10, *p* = .078, η_p_^2^ = 0.08. See the Supplementary Material Document for more details on this analysis.

### 3.4 The psychological and physiological association

#### 3.4.1 Cortisol

**Fig. 8:**
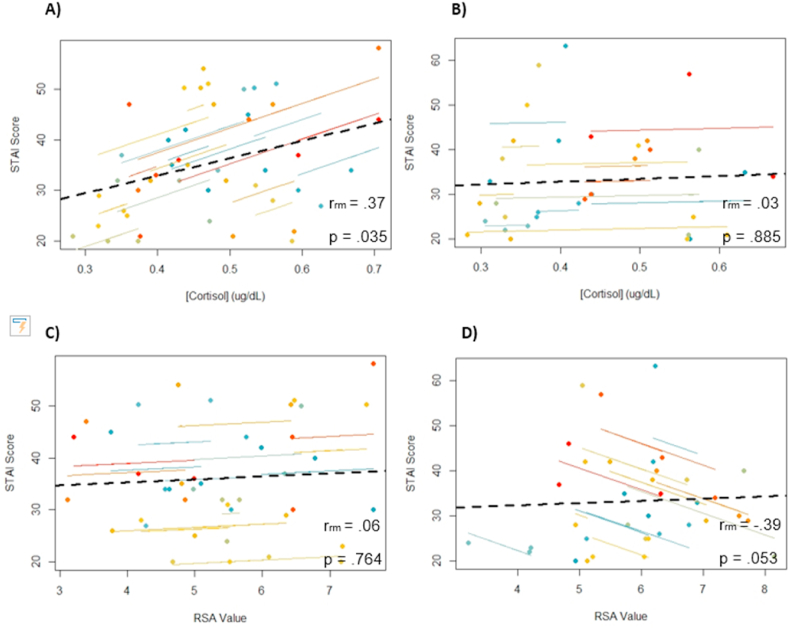
*Repeated-measure correlations between STAI-SA at T*_*1*_*, T*_*3*_*, T*_*8*_*and cortisol concentration at T*_*2*_*, T*_*4*_*, T*_*8*_*for A) the NoMI group and B) the MI group and repeated-measure correlation between STAI-SA at T*_*1*_*, T*_*3*_*, T*_*8*_*and RSA at T*_*2*_*, T*_*4*_*, T*_*8*_*for C) the NoMI group and D) the MI group*.

We performed a repeated-measures correlation between STAI-SA at T_1_, T_3_, T_8_, and cortisol at T_2_, T_4_, and T_8_. Analysis revealed no significant correlation for the whole sample, *r*(55) = 0.20, *p* = .14, CI 95% [-0.07, 0.44]. When evaluating MI values separately, correlation between these variables was also not significant, *r*(23) = 0.03, *p* = .89, CI 95% [-0.39,.44]. Notably, increased STAI-SA was related to an increase in cortisol in the NoMI group, *r*(31) = 0.37, *p* = .035, CI 95% [0.02, 0.64] (see Fig. 8).

Similar repeated-measures correlations established in the complete sample using the cortisol values and positive affect scores on the PANAS revealed the two measures to be positively correlated, *r*(55) = 0.37, *p* = .004, CI 95% [0.12, 0.58]. However, group-specific correlations failed to reach significance [MI group, *r*(23) = 0.22, *p* = .29, CI 95% [-0.21, 0.58]; NoMI group, *r*(31) = 0.53, *p* = .002, CI 95% [0.21, 74]]. As for negative affect on the PANAS, the results of repeated measures correlations were not significant for the whole sample, *r*(55) = −0.19, *p* = .16, CI 95% [-0.43, 0.08] nor for the MI group, *r*(23) = 0.05, *p* = .80, CI 95% [-0.37, 0.45]. A negative correlation was found between cortisol and negative affect in the NoMI group, *r*(31) = −0.38, *p* = .029, CI 95% [-0.65, −0.03].

#### 3.4.2 HRV

We tested the possible relationship between subjective emotional response and RSA. Repeated-measures correlations between STAI-SA at T_1_, T_3_, T_8_ and RSA (at 5 min pre T_2_, at 5 min pre T_3_, and 5 min pre T_8_), making the RSA assessments coincident with the time at which participants filled the STAI and PANAS questionnaires. The correlation was not significant between STAI-SA and RSA in the whole sample, *r*(51) = −0.15, *p* = .30, CI 95% [-0.41, 0.13], nor the NoMI group, *r*(27) = 0.06, *p* = .76, CI 95% [-0.33, 0.43], but close to significance for the MI group, *r*(23) = −0.39, *p* = .053, CI 95% [-0.69, 0.02] (see Fig. 8). A negative correlation would follow the expected pattern: Increased subjective stress co-occurring with reduced parasympathetic control. No other significant correlations were found.

## 4 Discussion

This pilot study aimed to characterize and compare various physiological outcomes (i.e., cortisol, HRV) measured during basal and stress-induced conditions and characterize psychological response profiles (using STAI-SA and PANAS) of MI women and a matched control group. At present, no research has monitored in vivo the acute changes in stress-related physiological outcomes associated with social stress exposure in women with a history of MI using the TSST.

### 4.1 Psychological changes

We used the STAI-TA and -SA versions to measure trait and state anxiety, respectively [56], and the PANAS to measure positive and negative affect [58]. Participants completed the STAI-SA and PANAS at three critical intervals during the study (i.e., arrival to the laboratory [T_1_], after the TSST [T_3_], and at the end of the study [T_8_]). We found no difference between the MI and NoMI groups for the STAI-Trait Anxiety (STAI-TA), which indicates similar daily life anxiety profiles in both groups. However, participants’ scores on the STAI-10.13039/100012106SA support an effect of the TSST to increase anxiety levels in both groups. Specifically, anxiety levels increased significantly at T_3_ and T_8_ compared to T_1_, and a significant decrease is noted between T_3_ and T_8_. In addition to increased anxiety related to TSST exposure, participants' affect gained in positivity as the experiment progressed from pre-to post- TSST, with negative affect decreasing over the same period. Specifically, we found the T_3_ measure, collected post TSST, to be associated with scores supporting increased positive affect compared to measures taken at T_1._ Consistent with this, as participants recovered from TSST exposure, scores indicated significantly reduced negative affect at both T_3_ and T_8_ compared to T_1_. Overall, these results suggest that participants entered the study with elevated anxiety levels, which levels gradually decreased throughout the study, with a peak negative emotionality related to TSST exposure. Considering that participants had to complete a battery of neuropsychological tests after the TSST, which can be stressful, our findings support participants to have acclimatized to the anxiety-provoking environment. Our observations are consistent with the TSST being a validated stressful paradigm that significantly activates the endocrine secretion of cortisol [54,71,72]. Our study also suggests that performing neuropsychological assessments, possibly due to individual test completion, likely made participants feel an increased control compared to the TSST.

### 4.2 Physiological changes

Eight cortisol samples were collected at key intervals during the study [54], and HRV was continuously recorded. We did not find significant between-group alteration in HRV; however, *p*-values and effect sizes suggest that this could be attributable to statistical power related to a small sample. Although the cortisol response of the MI group appeared on average tempered compared to measures of the NoMI group, no significant group differences were detected. Follow-up analyses on individual time intervals, enabled by priory hypotheses, revealed lower cortisol levels in MI compared to NoMI participants at T_8_, with a trend emerging at T_7_.

We expected greater differences in cortisol secretion between MI and NoMI participants following TSST exposure. Indeed, the impact of cardiac arrest and associated hypoxic state on HPAA reactivity has been long recognized [41,42,[73], [74], [75]], as well as the impact on brain tissues and systems associated with this response [37,[76], [77], [78], [79], [103]]. In this context, Zhao et al. [37] recently demonstrated resuscitation from cardiac arrest in mice to significantly increase pro-inflammatory cytokines secretion, promoting elevated HPAA activation and glucocorticoid secretion upon stress exposure. The authors also noted significant atrophy of lymphoid organs dimensions, which further impacted HPAA function. Although of reduced magnitude, MI impacts brain oxygenation and induces damage to the hippocampus, which regulates HPAA activation [80,81]. Thus, MI represents a potent metabolic stressor, likely to have repercussions on stress-induced physiological (i.e., heart rate and cortisol secretion) and emotional reactivity.

Our study is the first to examine how MI in women impacts HPAA response upon exposure to a social stressor. Jackson et al. [31] characterized 221,677 individuals' levels of psychological distress and risk for MI over 5 years. Authors found that participants who experienced an MI were those who reported high/very high psychological distress, and that this factor increased the risk of experiencing a second MI by 20.0%. Similarly, Roest et al. [82] reported that individuals experiencing high levels of post-MI anxiety were 36.0% more likely to have cardiac complications (e.g., mortality, risk of having another MI). Considering that SCA and MI have a similar impact on the brain (i.e., lack of oxygen and nutrients), although, at a different degree, the effect of MI on brain functions may be more subtle. Nevertheless, with an increased occurrence of mental health disorders post-MI related to heightened mortality rate, MI may play a significant role in HPAA functionality of individuals showing elevated psychological distress post-MI [31,80,82]. Nonetheless, our findings suggest that MI could be associated with reduced flexibility in the stress response over a long period. Follow-up studies will need to investigate this in larger sample sizes, where psychological distress would act as a covariate.

### 4.3 The relation between physiological and psychological changes

Despite a small sample, our findings partially support the idea that there is a disconnect between subjective (e.g., how I feel) and objective experience for MI participants, particularly for cortisol response. At a group level, this is illustrated by the fact that even though the MI and NoMI groups perceived the TSST as a stressful experience, the physiological response to stress tended to be slightly larger for the NoMI than MI group overall and significantly so towards the end of the experiment for cortisol. At the individual level, we observed strong and significant correlations between changes in subjective experience (e.g., anxiety, positive and negative affect) and cortisol levels only in the NoMI group and not in the MI group. These findings support that the HPAA is not properly adapting in its response to stressful events, particularly when prolonged, possibly due to an altered negative feedback mechanism [83,84]. Future studies should include more than one marker of stress, as women with a MI history might be more attuned to cardiovascular indices of stress, as suggested by the repeated correlations between subjective anxiety and RSA. In addition, it is essential to highlight that psychophysiological response depends on sex, and it is, therefore, necessary to consider this factor in the analyses and interpretations of the collected findings [85].

## 5 Limits of the study

The global pandemic did not allow the recruitment of additional participants, which could be tested under similar basal conditions. Our sample size did not enable accounting the effect of diabetes on our physiological data (i.e., CORT and HRV). However, future studies should consider this condition as it is known to impact HRV data (for more information, see Ref. [86]). In addition, the sample size of our study prevented addressing possible effects of the participants’ prescribed medication on the measured physiological responses. Therefore, one cannot rule out the impact of post MI medication on some responses. Such effects however remain difficult to determine as influence of medical treatments likely depend on post MI recovery period.

Considering our observations, we believe that replicating our study with a bigger sample would refine our findings and statistical tendencies observed to be validated. It also appears necessary to assess multiple correlates of stress (e.g., cortisol, electrodermal activity, heart rate), especially in women, because their reaction is different than men, and not all tools may be sensitive enough to capture these differences [[87], [88], [89]]. In addition, adding two supplementary groups that would use the friendly-TSST to compare the difference between stress (TSST) and non-stress (friendly-TSST) would allow capturing a global picture of the effect of the HPAA. Indeed the non-stressful situation would allow researchers to compare how stress impacts physiological activation (i.e. HPAA) and perception of stress with the stressful situation. Without a doubt, studies also need to include participants from different racial/ethnic background as the HPAA respond differently in BIPOC population [[90], [91], [92], [93], [94], [95]]. Finally, future studies should also include measures of Type D personality - disposition to repress emotional distress [96] - as it has been shown that individuals with CHD are more prone to have this type of presentation, which could potentially explain the differences in perceived stress (e.g., mental distress; [[97], [98], [99], [100]], and might impact the prognosis in MI individuals [101,102].

## 6 Conclusion

This pilot research is the first to characterize stress-induced changes in HPAA activation via cortisol secretion in women with MI history. Despite a small sample, we found that women with MI reported similar levels of perceived stress compared to the control group; however, the different physiological measures collected indicated a psychophysical dissonance in the response profile of MI women, especially as it pertained to RSA and cortisol secretion, which appeared attenuated post-stress in MI women. Our findings open the door to a more in-depth examination of the parameters of MI recovery that are most closely associated with altered cardiovascular and HPAA responses, since dysregulation of such responses could explain why some people present an increased risk of developing mental health disorders following MI.

## CRediT author statement

Conceptualization: N.F.N.L., H.P.

Methodology: N.F.N.L. K. M-S., A.F.N.T.

Formal Analysis: N.F.N.L., K.M-S., A.F.N.T.

Investigation: N.F.N.L.

Resources: H.P.

Data Curation: N.F.N.L, K. M-S., A.F.N.T.

Writing - Original Draft: N.F.N.L., K.M-S., A.F.N.T., H.P.

Writing - Review & Editing: N.F.N.L., K.M-S., A.F.N.T., H.P.

Visualization: N.F.N.L., K.M-S., A.F.N.T.

Supervision: H.P.

Project Administration: N.F.N.L.

Funding acquisition: H.P.

## Declarations of competing interest

None.

## Footnotes

2 Female and woman are two distinctive terms and not interchangeable. While sex refers to the biological characteristics at birth, gender refers to an individual's expression, identity, and societal roles. Consequently, when the term woman is used in this article, we will refer to the biological characteristics.
