# Supplementary material for: The impact of myocardial infarction on basal and stress-induced heart rate variability and cortisol secretion in women: A pilot study
Source: Compr Psychoneuroendocrinol. 2022 Jan 13;9:100113. doi: 10.1016/j.cpnec.2022.100113 (PMC9216611; doi:10.1016/j.cpnec.2022.100113)
Supplement: Multimedia component 2 [file mmc2.docx]

**Supplementary material**

### *2.5.1 Trier Social Stress Test*

As for the TSST protocol, participants had 5 minutes to prepare for a speech task and were given a piece of paper to write their thoughts. However, they were instructed that their notes could not be used during the speech delivery. Then, participants had to stand up and deliver a 5-minute oral presentation telling the jury why they would be the best candidate for their dream job. Participants were encouraged to continue presenting, and if they experienced difficulties, a series of standardized questions were asked to enhance continued speech. Finally, participants completed the arithmetic task for 5 minutes which entailed subtracting 17 from 2040 until 0. Every time the participant made a mistake, she was instructed to start from the beginning. The panel was composed of two undergraduate students (a woman and a man) trained to deliver the TSST protocol. These judges wore lab coats and maintained a neutral expression through the duration of the TSST. Before initiating the speech task, one judge (man) stated to the participant that three cameras – he pointed to them - were going to record their performance since both judges were 'behavioural analysis' experts; the judge also indicated that they were there going to take notes. During the debriefing, N.F.N.L. shared that no recording was performed, but that such mention participated in creating a stressful environment.

2.5.3.2 Heart Rate Variability

We visually inspected all data for incorrectly identified peaks, missing heartbeats, and any portion of data that would be unusable due to excessive noise. We interpolated up to two beats when beats were missing or diverged substantially from their expected time. We removed incorrect R peaks and inserted the correct R peaks manually when these were clear. Additionally, we discarded data portions with excessive noise, but all included segments contained at least 30 seconds of continuous data. After cleaning, we excluded the data of one MI and one NoMI participants from HRV analyses because they retained a low percentage of normal peaks (i.e., not estimated, not ectopic; mean of 82.04% and 87.61%, respectively). All other participants had more than 90% of normal R peaks in each segment (sample mean of 99.29%, *SD* = 1.58). The respiratory peak frequency of all segments was within the RSA frequency band, which was defined as 0.15 to 0.40 Hz.

Portions of the collected data were selected for analysis. Thus, segments of 5 minutes heart rate recordings were analyzed related to each of the collection intervals T_1_ to T_7_. Further, as changes related to stress induction was of importance, the entirety of the TSST was analyzed by segmenting the data between T_2_ (beginning of TSST) and T_3_ (end of TSST). Lastly, HRV reported for T_8_ represents the 300-second preceding initiation of this time interval (this period corresponds to the end of neuropsychological testing). Similarly, T_2_ represents HRV measured 300-second before initiation of this time interval and evaluate baseline.

## 2.6 Statistical Analyses

The first set of analyses compared the NoMI and MI groups on psychological tests (i.e., STAI and PANAS) using mixed ANOVAs. The two groups were further compared on trait anxiety with independent samples t-test, 5000 bootstraps, and bias-corrected accelerated confidence intervals, like all other t-tests reported here.

The second set of analyses compared the NoMI and MI groups on objective measures of stress, first cortisol and then respiratory sinus arrhythmia (RSA), using mixed ANOVAs and independent samples t-tests. In addition to comparing cortisol throughout the experiment, we also compared the response to the TSST, using the area under the curve to ground (AUC_G_) and to increase (AUC_I_; Pruessner et al., 2003), and peak reactivity (Khoury et al., 2015). We considered T_2_ the 'baseline' for AUC_G_, AUC_I_, and peak reactivity because it was collected after the habituation period and before the TSST (similar to RSA analyses, described below). We selected this period as the baseline as it represented when the participants were most relaxed (i.e., they relaxed by sitting down and reading a text of their choice or watching a calming video). To calculate AUC_G,_ we obtained the sum of the mean cortisol of two subsequent periods (T_2_ and T_3_, T_3_ and T_4_, T_4_ and T_5_, T_5_ and T_6_), with each means being multiplied by that period's duration in minutes (Pruessner et al., 2003). AUC_I_ was identical to AUC_G_ but entailed subtracting the T_2_ value multiplied by the total time between T_2_ and T_6_ (Pruessner et al., 2003). Peak reactivity was defined as each participants' highest cortisol value from T_3_ to T_6_ subtracted from their own T_2_ value (Khoury et al., 2015).

We ran separate mixed ANOVAs to compare the two groups on RSA values extracted over the experiment and over the entire TSST period. Participants' RSA value for the 'baseline' period (5 minutes pre T2) was subtracted from each of these segments. Hence, a negative value reflects reduced parasympathetic control relative to baseline – consistent with a stress response from the parasympathetic system – and positive values represent higher parasympathetic control relative to baseline.

Lastly, we performed repeated-measures correlations (Bakdash & Marusich, 2017) to test the within-subject relation between subjective (i.e., STAI, PANAS) and objective measures (i.e., cortisol, RSA) across three timestamps during the experiment. These correlations included the whole sample in a first step and were repeated in each group as we anticipated that subjective experience would align less well to objective experience in the MI than the NoMI group.

One participant from the MI group missed a cortisol value for T_1_, and another participant missed a T_1_ segment for RSA analyses. They were excluded whenever that particular T_1_ measurement was included in the analyses. For the questionnaires, the mean value for each item of the whole sample was given if a participant forgot to fill out an item (STAI-SA_T1_ = 4; STAI-SA_T3_ = 1; STAI-TA = 1; PANAS_T8_ = 4 participants).

### *3.3.1 Cortisol*

The AUC_G_ was not significantly reduced in MI (*M* = 14.23, *SE* = 1.36) compared to NoMI (*M* = 16.24, *SE* = 1.56) group. A between group mean difference of 2.02, BCa 95% [-2.09, 6.25], *t*(27) = 0.98, *p* = .34, Hedges’ *g* = 0.36, CI 95% [-0.39, 1.09] was detected. Similarly, the AUC_I_ was not significantly reduced in the MI (*M* = -0.05, *SE* = 0.97) compared to NoMI (*M* = 0.80, *SE* = 0.76) group. A between group mean difference of 0.84, BCa 95% [-1.44, 3.23], *t*(27) = 0.70, *p* = .49, Hedges’ *g* = 0.25, CI 95% [-0.47, 0.96] was detected. Finally, the MI (*M* = 0.06, *SE* = 0.02) and NoMI group (*M* = 0.08, *SE* = 0.02) groups showed comparable peak reactivity, with mean difference of 0.02, BCa 95% [-0.03, .07], *t*(27) = 0.73, *p* = .48, Hedges’ *g* = 0.26, CI 95% [-0.46, 0.97].

### *3.3.2 Heart Rate Variability*

We expected the TSST to induce differential impact on heart rate stress-related response. The initial analysis only included a portion of the TSST (i.e., speech preparation). Our follow up analysis included the complete TSST exposure period. The mixed ANOVA included Group (MI, NoMI) as a between-subject factor and three periods of 5 minutes for Time (speech preparation, speech delivery, arithmetic task) as a within-subject factor (Figure 7). Neither the main effects nor the interaction were significant: Time, *F*(2, 50) = 1.90, *p* = .16, η_p_^2^ = .07; Group, *F*(1, 25) = 0.93, *p* = .344, η_p_^2^ = .04, Time*Group, *F*(2, 50) = 2.23, *p* = .12, η_p_^2^ = .08.

**References**

Bakdash, J. Z., & Marusich, L. R. (2017). Repeated measures correlation. *Frontiers in Psychology*, *8*, 456. https://doi.org/10.3389/fpsyg.2017.00456

Khoury, J. E., Gonzalez, A., Levitan, R. D., Pruessner, J. C., Chopra, K., Basile, V. S., Masellis, M., Goodwill, A., & Atkinson, L. (2015). Summary cortisol reactivity indicators: Interrelations and meaning. *Neurobiology of Stress*, *2*, 34–43. https://doi.org/10.1016/j.ynstr.2015.04.002

Pruessner, J. C., Kirschbaum, C., Meinlschmid, G., & Hellhammer, D. H. (2003). Two formulas for computation of the area under the curve represent measures of total hormone concentration versus time-dependent change. *Psychoneuroendocrinology*, *28*(7), 916–931. https://doi.org/10.1016/S0306-4530(02)00108-7
